# Licoflavone B mitigates allergic rhinitis by restraining epithelial PI3K–AKT/NF-κB NLRP3 inflammasome signaling

**DOI:** 10.1007/s13577-026-01456-3

**Published:** 2026-11-05

**Authors:** Qi Shen, Xiaoyao Ma, Hui Liu, Xuemin Li, Wenhui Shao

**Affiliations:** Department of Otorhinolaryngology, The First Affiliated Hospital of Henan University of Chinese Medicine, No. 19, Renmin Road, Zhengzhou, 450000 Henan China

**Keywords:** Licoflavone B, Allergic rhinitis, Nasal epithelium, PI3K–AKT, NF-κB, NLRP3 inflammasome, Cytokines, IGF-1 rescue, Poly(i:c), RNA-seq, Molecular docking

## Abstract

Allergic rhinitis remains highly prevalent and is often incompletely controlled by current therapies, while accumulating evidence implicates epithelial PI3K–AKT/NF-κB-mediated activation of the NLRP3 inflammasome in upper-airway inflammation. This study investigated whether the licorice-derived flavonoid Licoflavone B (LFB) alleviates allergic rhinitis by modulating this signaling axis. In an ovalbumin-induced murine model, allergic rhinitis-like symptoms and nasal histopathological changes were evaluated, cytokine levels were quantified by ELISA, and the activation of the NLRP3 inflammasome and PI3K–AKT/NF-κB signaling was assessed by immunoblotting. Unbiased RNA sequencing followed by KEGG and GO enrichment analyses was performed to identify potential upstream pathways. The involvement of PI3K–AKT/NF-κB signaling was further explored through PI3K–AKT reactivation with IGF-1 and TLR3 stimulation with poly(I) in vivo and in primary human nasal epithelial cells (HNEpCs). Molecular docking was also conducted to explore the potential interaction between LFB and PI3K. LFB reduced sneezing and nasal scratching, partially ameliorated epithelial thickening and inflammatory-cell infiltration, and decreased mast-cell staining. LFB also reduced the levels of IL-1β, IL-6, IL-5, IL-13, and TNF-α, as well as the expression of NLRP3-inflammasome components. RNA-sequencing analysis identified PI3K–AKT signaling as one of the major enriched pathways, and immunoblotting confirmed that LFB decreased the phosphorylation of PI3K, AKT, and p65 without substantially altering their total protein levels. In HNEpCs, IGF-1 restored pathway phosphorylation and partially reinstated cytokine and inflammasome protein expression, thereby attenuating the effects of LFB. Similarly, IGF-1 or poly(I) partially diminished the symptomatic and histological improvements induced by LFB in vivo. Molecular docking positioned LFB within the PI3K catalytic pocket, providing structural plausibility for a potential LFB–PI3K interaction. Collectively, these findings indicate that LFB alleviates ovalbumin-induced allergic rhinitis-like inflammation and is associated with reduced activation of the PI3K–AKT/NF-κB/NLRP3 signaling axis in nasal tissues, HNEpCs, and stimulated mast-cell models. The partial reversal of these effects by IGF-1 or poly(I) further supports the involvement of this signaling pathway.

**Supplementary Information:**

The online version contains supplementary material available at https://doi.org/10.1007/s13577-026-01456-3.

## Introduction

Allergic rhinitis (AR) is a common, immune-mediated disorder of the upper airway that impairs quality of life and productivity and remains underdiagnosed and undertreated worldwide [1, 2]. Population-based estimates place rhinitis prevalence in the range of ~10–30% globally, with substantial regional variability and healthcare burden [3]. Current management—intranasal corticosteroids, oral antihistamines, leukotriene antagonists, and allergen immunotherapy—controls symptoms for many patients but is limited by incomplete responses, adherence issues, and recurrence; moreover, these approaches do not directly target inflammasome-centric mechanisms now implicated in airway disease [4–6].

Mounting evidence indicates that the NLRP3 inflammasome contributes to type−2–skewed upper-airway inflammation and mucosal remodeling in experimental and clinical settings, positioning NLRP3 as a therapeutic node[7, 8]. Upstream of NLRP3, epithelial and myeloid PI3K–AKT–NF-κB signaling integrates allergen and viral-mimetic cues to amplify cytokine production [9, 10]; PI3K activity in airway epithelium can drive NLRP3-dependent inflammation, and NF-κB stimulation with poly(I:C) robustly elicits epithelial cytokines relevant to rhinitis pathobiology[11, 12]. These axes provide tractable readouts for pathway-focused intervention and mechanistic validation.

Natural-product-derived small molecules have attracted increasing attention as potential anti-inflammatory therapeutics because of their structural diversity, multitarget activities, and generally favorable safety profiles [13, 14]. In particular, flavonoids isolated from licorice and other medicinal plants exhibit broad anti-inflammatory and immunomodulatory effects in multiple inflammatory disease models [15, 16]. Previous studies have shown that several flavonoids can suppress NF-κB activation, inflammasome signaling, oxidative stress, and cytokine production, highlighting their potential relevance in airway inflammatory diseases [17]. Nevertheless, the effects of specific flavonoid monomers on allergic rhinitis, especially in the context of inflammasome-associated signaling pathways and epithelial–mast cell inflammatory networks, remain insufficiently understood.

Licoflavone B (LFB) is a naturally derived flavonoid compound isolated from licorice with reported anti-inflammatory and immune-regulatory potential [18]. However, whether LFB can modulate allergic airway inflammation and its associated signaling mechanisms has not been systematically investigated. Based on the emerging role of the PI3K–AKT/NF-κB/NLRP3 axis in AR pathogenesis and the growing interest in flavonoid-mediated immune regulation, we hypothesized that LFB may attenuate allergic rhinitis by suppressing inflammasome-associated inflammatory signaling.

Therefore, in the present study, we investigated the effects of LFB in experimental models of allergic rhinitis by integrating behavioral and histopathological evaluation with cytokine and chemokine profiling, inflammasome analysis, pathway-reactivation experiments, and molecular docking approaches. In particular, we focused on determining whether the anti-inflammatory effects of LFB are associated with modulation of the PI3K–AKT/NF-κB/NLRP3 signaling cascade in nasal epithelial cells and mast cells.

## Methods and materials

### Reagents and antibodies

Licoflavone B (LFB; analytical grade, ≥98%; MedChemExpress, Cat. HY-N4184) was prepared per vendor instructions; poly(I:C) HMW (InvivoGen, Cat. tlrl-pic) served as the TLR3 agonist; recombinant human IGF-1 (PeproTech, Cat. 100–11) was reconstituted immediately before use. For immunoblotting, the following primary antibodies were used: NLRP3 (Cell Signaling Technology, #15,101), ASC/TMS1 (CST, #13,833), caspase−1 (CST, #2225), IL-1β—mouse specific (CST, #8689), phospho-PI3K p85 (Tyr458)/p55 (Tyr199) (CST, #4228), total PI3K p85 (CST, #4292), phospho-AKT (Ser473) (CST, #4060), total AKT (pan) (CST, #4691), phospho-NF-κB p65 (Ser536) (CST, #3033), total NF-κB p65 (CST, #8242), and GAPDH (CST, #2118); HRP-conjugated anti-rabbit IgG (CST, #7074) and anti-mouse IgG (CST, #7076) were used as secondaries. Cytokines were quantified with mouse ELISA kits (Invitrogen/eBioscience): IL-1β (88–7013-88), IL-6 (88–7064-88), IL-5 (88–7054-88), IL-13 (88–7137-88), and TNF-α (88–7324-88), all validated by the manufacturer for tissue homogenates.

### Animals and induction of AR

Male BALB/c mice (6–8 weeks; SPF) were acclimatized under a 12 h light/dark cycle (22–24 °C; 40–60% humidity) with food and water ad libitum. Allergic rhinitis (AR) was induced by i.p. sensitization with ovalbumin (OVA; 50 µg) emulsified in aluminum hydroxide (1 mg) on days 0 and 7, followed by intranasal challenges with 1% OVA (20 µL/nostril) once daily on days 14–20 under light isoflurane anesthesia. Control mice received alum/vehicle sensitization and saline intranasal challenge.

### LFB formulation and in vivo administration; pathway reactivation

LFB was prepared as a DMSO stock and diluted in sterile PBS (final DMSO ≤0.5%). Unless indicated, mice received LFB 25 mg·kg⁻^1^ by oral gavage 1 h before each intranasal challenge on days 14–20. For pathway or innate reactivation, mice additionally received IGF-1 (1 mg·kg⁻^1^, i.p., 30 min before LFB) or poly(I:C) (high-molecular-weight; 1 mg·kg⁻^1^, intranasal co-instillation with OVA). Experimental groups: OVA, OVA +LFB, OVA +LFB+IGF-1, OVA +LFB+poly(I:C).

### Behavioral symptom scoring

Immediately after the final challenge, sneezing and nasal scratching episodes were recorded for 10 min by two blinded observers; counts were averaged per mouse.

### Tissue collection and histology

Mice were euthanized 24 h after the last challenge. Heads were fixed in 4% paraformaldehyde, decalcified in 10% EDTA (pH 7.4, 10–14 days, 4 °C), paraffin-embedded, and coronally sectioned (4 µm). Sections were stained with hematoxylin and eosin (H&E) to evaluate epithelial thickness, edema, and inflammatory infiltration (semi-quantitative scoring by two blinded pathologists). Mast cells were visualized by toluidine blue (0.1%, pH 2.3, 2–3 min); metachromatic cells in the lamina propria were counted in ≥5 high-power fields (HPF) per section.

### Cytokine quantification (ELISA)

Nasal mucosa was micro-dissected on ice, homogenized in PBS with protease inhibitors, and clarified (12,000×g, 10 min, 4 °C). IL-1β, IL-6, IL-5, IL-13, CCL2, CXCL12 and TNF-α were quantified using mouse ELISA kits per manufacturer’s instructions. Standard curves were run on each plate; samples were assayed in duplicate.

### Protein extraction and immunoblotting

Tissues or cells were lysed in RIPA buffer supplemented with protease/phosphatase inhibitors, clarified, and quantified (BCA). Equal protein (20–40 µg) was resolved by SDS-PAGE and transferred to PVDF membranes. After blocking (5% BSA), membranes were incubated overnight (4 °C) with primary antibodies to NLRP3, ASC, caspase−1, IL-1β, phospho-PI3K (p85 Tyr458/p55 Tyr199), total PI3K, phospho-AKT (Ser473), total AKT, phospho-p65 (Ser536), total p65, and GAPDH, followed by HRP-conjugated secondary antibodies. Bands were developed by chemiluminescence and quantified by densitometry (ImageJ), normalized to GAPDH or to total protein for phospho/total pairs.

### CCK-8 assay

Cell viability was measured using the Cell Counting Kit-8 (CCK-8; WST-8 colorimetric assay) in 96-well flat-bottom plates. Human nasal epithelial cells (HNEpCs, ATCC) in logarithmic growth were dissociated, counted, and seeded at 5×103 cells/well in 100 µL complete medium. After 12–16 h attachment at 37 °C, 5% CO₂, culture medium was replaced with treatment media as indicated (e.g., LFB, IGF-1, poly(I:C)); the vehicle control contained ≤0.1% DMSO. Blank wells (medium + CCK-8, no cells) and an optional positive control (e.g., staurosporine, 0.5–1 µM) were included. Following 24–48 h treatment, 10 µL CCK-8 reagent (10% v/v) was added directly to each well and incubated 60–120 min at 37 °C, protected from light. Absorbance was read at 450 nm (reference 650 nm) using a microplate reader.

### In-vitro stimulation, LFB treatment, and pathway rescue in HNEpCs

Confluent HNEpCs were pre-treated with LFB (10–25 µM, 1 h) and stimulated with IL-4/IL-13 (10 ng mL⁻^1^ each) with or without poly(I:C) (10 µg mL⁻^1^) for 6–24 h. Supernatants were collected for ELISA; cell lysates were processed for immunoblotting.

### Mast-cell culture, activation, and treatment

RBL-2H3 cells, a rat basophilic leukemia cell line commonly used as a mast-cell model, were obtained from Cmbio and cultured in Dulbecco’s modified Eagle’s medium (DMEM) supplemented with 10% fetal bovine serum (FBS) and 1% penicillin–streptomycin at 37 °C in a humidified incubator containing 5% CO₂.

For mast-cell activation, RBL-2H3 cells were seeded into 6-well plates and allowed to adhere overnight. The cells were sensitized with anti-DNP IgE (0.5 μg/mL) for 12 h, gently washed with PBS, and then stimulated with DNP-HSA (100 ng/mL) for 24 h to induce mast-cell inflammatory activation. To evaluate the effect of LFB, cells were pretreated with LFB (25 μM) for 1 h before DNP-HSA stimulation. For pathway rescue experiments, IGF-1 (100 ng/mL) was added 1 h before LFB treatment and maintained during subsequent stimulation, according to the experimental design. After stimulation, culture supernatants were collected for ELISA analysis of inflammatory cytokines, including IL-1β, IL-5, IL-6, IL-13, and TNF-α. Cell lysates were collected for western blot analysis of NLRP3 inflammasome-related proteins, including NLRP3, ASC, caspase−1, and IL-1β, as well as PI3K–AKT/NF-κB pathway-related proteins, including PI3K, p-PI3K, AKT, p-AKT, p65, and p-p65. Each in vitro experiment was independently repeated at least three times.

### RNA extraction, library construction, and RNA-seq

Total RNA from nasal mucosa (n ≥ 3/group) was extracted with TRIzol, DNase-treated, and quality-assessed (Agilent Bioanalyzer; RNA Integrity Number ≥7). Poly(A)-selected, strand-specific libraries (Illumina TruSeq) were sequenced (paired-end 150 bp) on an Illumina platform (≥ 30 M read pairs/sample). Reads were trimmed (fastp), aligned to the mouse genome (GRCm39) with STAR, and counted with featureCounts. Differential expression was performed using DESeq2 with Benjamini–Hochberg correction; differentially expressed genes (DEGs) were defined as |log₂FC|≥ 1 and adjusted P < 0.05. Volcano plots and heatmaps were generated from normalized counts. DEGs were analyzed with clusterProfiler for KEGG and Gene Ontology (biological process, cellular component, molecular function) using FDR < 0.05. Leading-edge gene sets for PI3K–AKT were extracted for visualization and orthogonal validation by immunoblotting.

### Molecular docking

To contextualize pathway data, LFB was docked into the ATP-binding pocket of class I PI3K (human p110 catalytic domain). Proteins were prepared by protonation and removal of crystallographic waters/ligands; LFB was energy-minimized. Docking was performed using AutoDock Vina with a grid box centered on the catalytic site. Top-ranked poses were selected by binding energy and interaction geometry and visualized as 3D poses and 2D interaction maps. Docking was used as hypothesis-generating and not for affinity estimation.

### Statistics

Data are presented as mean ± s.d. unless stated. Normality was assessed by Shapiro–Wilk and homoscedasticity by Levene’s test. For ≥3 groups, one-way ANOVA with Tukey’s post-hoc test was used; for non-normal distributions, Kruskal–Wallis with Dunn’s correction was applied. Two-tailed α = 0.05. Exact n and replicate numbers are provided in figure legends. Significance annotations followed the legend conventions: * indicates comparison vs Control (or vs AR where specified); & indicates comparison vs AR (or vs AR+LFB where specified).

## Results

### LFB reduces AR-like behaviors and histopathology in the nasal mucosa

**Fig. 1:**
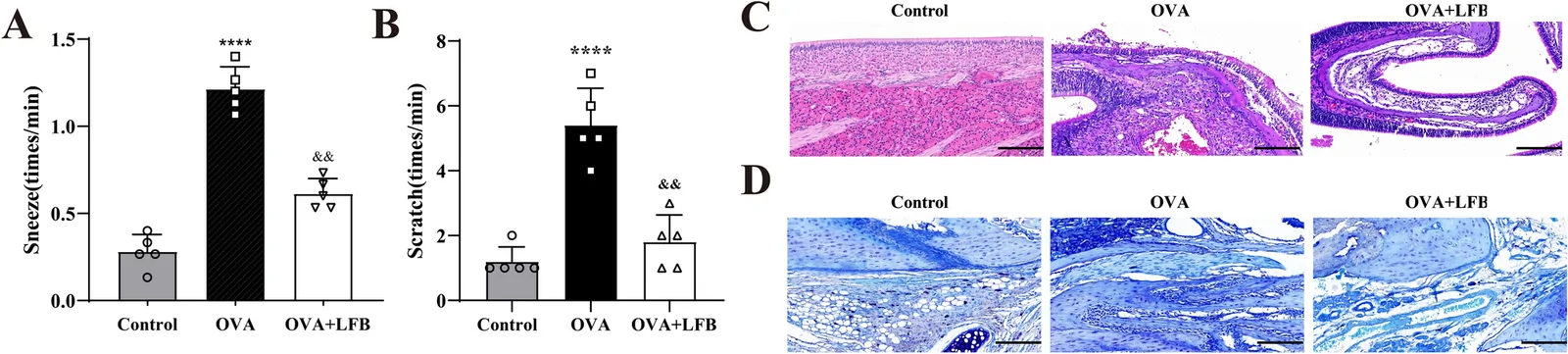
Licoflavone B (LFB) alleviates allergic-rhinitis–like symptoms and nasal mucosal pathology. **A** Sneezing frequency in each group. **B** Nasal scratching frequency in each group. Individual data points represent independent biological replicates (n = 5 mice per group). Data are presented as mean ± SD. **C** Representative H&E staining of nasal mucosa in each group. Scale bar = 50 μm. **D** Toluidine blue staining for mast cells in each group. Scale bar = 50 μm. Representative histological images were obtained from independent animals (*n* = 5 mice per group). *P< 0.05, **P< 0.01, ***P< 0.005, ****P< 0.001 versus control. ^&^*P*< 0.05, ^&&^*P*< 0.01, ^&&&^*P*< 0.005 versus OVA

To determine whether LFB confers phenotypic benefit in AR, we first quantified behavioral symptoms and nasal pathology. Compared with controls, AR mice exhibited pronounced symptomatology, including increased sneezing and nasal scratching (Fig. 1A,B). LFB treatment significantly decreased both behavioral readouts relative to the AR group, indicating symptomatic relief. Histologically, AR mucosa showed epithelial hyperplasia, edema, and dense inflammatory-cell infiltration on H&E, whereas LFB partially normalized epithelial architecture and reduced inflammatory infiltration (Fig. 1C). Consistently, toluidine blue staining revealed a marked expansion of mast cells in AR that was diminished by LFB (Fig. 1D). Together, these data demonstrate that LFB attenuates clinical signs and tissue pathology of AR.

### LFB attenuates airway inflammation via NLRP3-inflammasome restraint

**Fig. 2:**
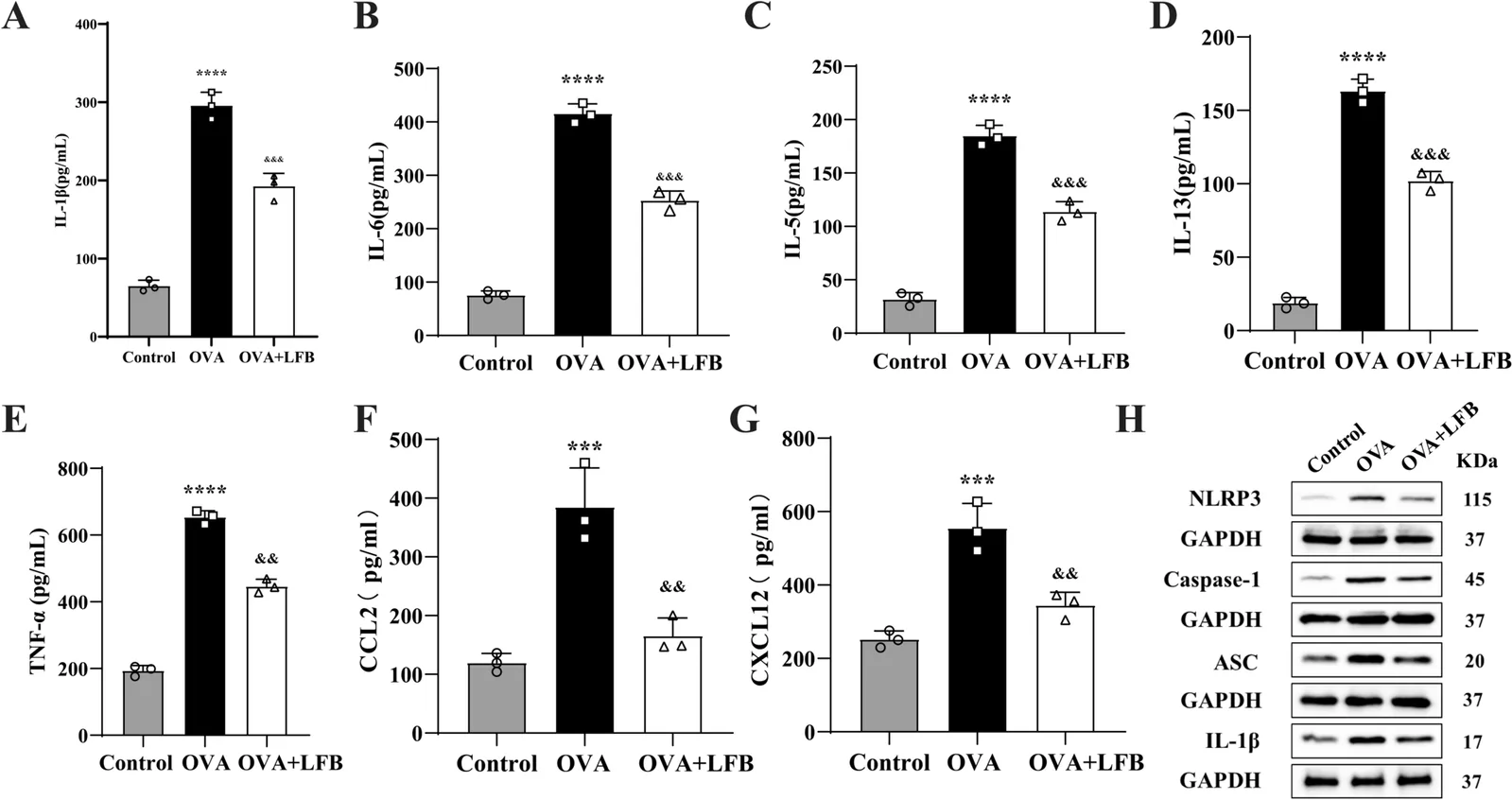
LFB dampens cytokine production and suppresses NLRP3-inflammasome activation. **A–G **ELISA quantification of IL-1β **(A)**, IL-6 **(B)**, IL-5 **(C)**, IL-13 **(D)**, TNF-α **(E)**, CCL2 **(F)** and CXCL12 **(G)** in the OVA model with or without LFB treatment. Individual data points represent independent biological replicates (n = 3 independent biological samples per group). Data are presented as mean ± SD. **H** Immunoblot analysis of NLRP3-inflammasome components in nasal tissues. Representative immunoblots were obtained from independent biological experiments performed in triplicate. *P< 0.05, **P< 0.01, ***P< 0.005, ****P< 0.001 versus control. ^&^*P*< 0.05, ^&&^*P*< 0.01, ^&&&^*P*< 0.005 versus OVA

Having established phenotypic improvement, we next asked whether LFB suppresses inflammatory mediators. Relative to controls, the AR group displayed robust increases in IL-1β, IL-6, IL-5, IL-13 and TNF-α (Fig. 2A–E). LFB treatment significantly decreased each cytokine compared with AR, indicating broad anti-inflammatory activity (Fig. 2A–E). In addition, ELISA analysis showed that CCL2 and CXCL12 levels were markedly elevated in AR nasal tissues, whereas LFB significantly reduced both chemokines (Fig. 2F,G). Consistently, immunoblotting revealed upregulation of NLRP3, ASC, caspase−1 and IL-1β in AR nasal tissues, whereas LFB reduced the abundance of these inflammasome components (Fig. 2H). These results support that LFB mitigates airway inflammation at least in part by restraining NLRP3-inflammasome signaling.

### LFB rewires the AR transcriptome and limits the PI3K–AKT/NF-κB axis

**Fig. 3:**
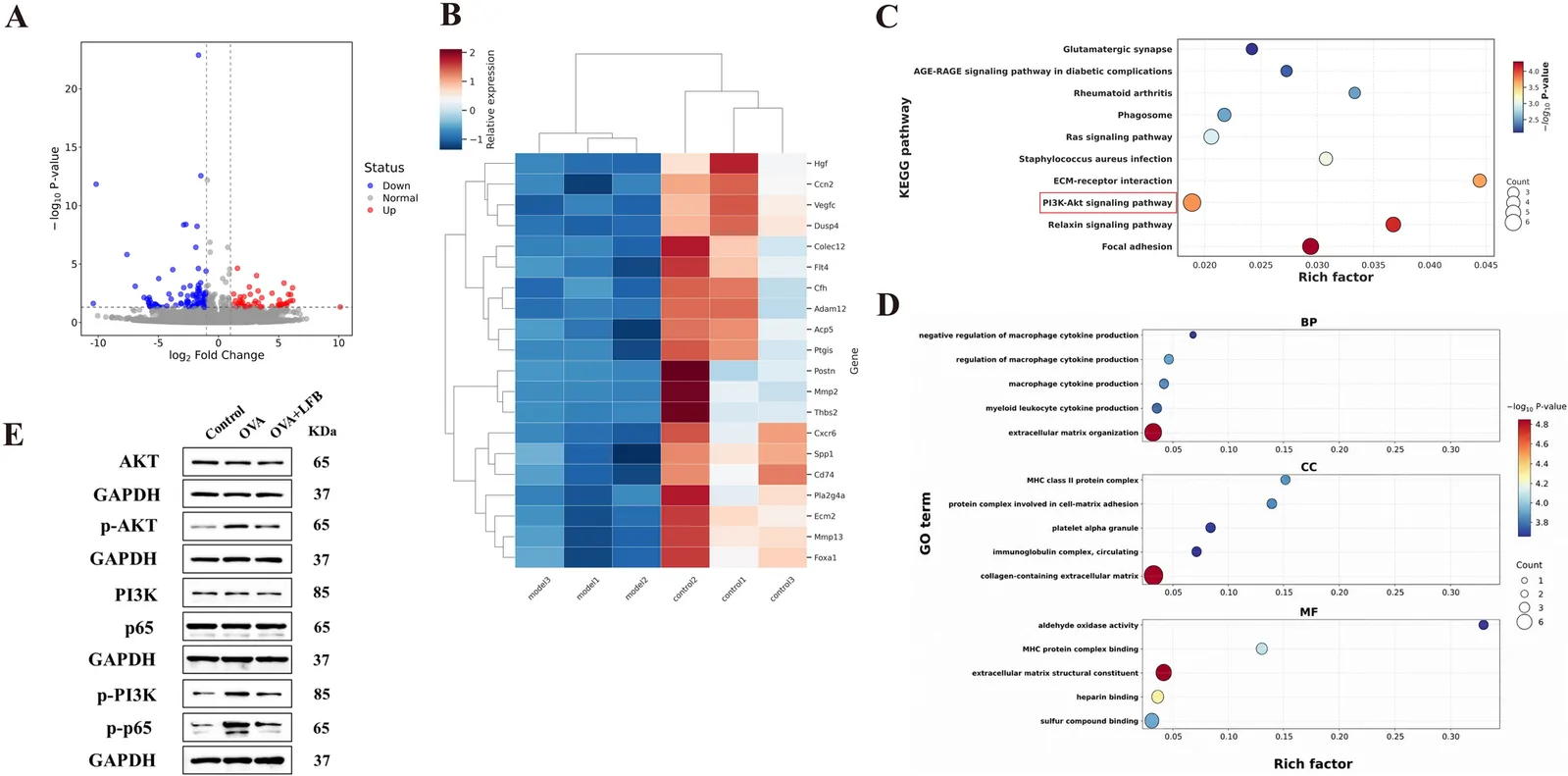
LFB reshapes the nasal mucosal transcriptome and restrains PI3K–AKT/NF-κB signaling. **A** Volcano plot of DEGs between OVA and LFB nasal mucosa. **B** Heat map of the top 20 representative DEGs. **C** The top 10 significantly enriched pathways in KEGG pathway enrichment analysis of DEGs. **D** GO enrichment analysis of DEGs (biological process, cellular component, molecular function). **E** Immunoblots validating PI3K–AKT/NF-κB pathway suppression. Representative immunoblots were obtained from independent biological experiments performed in triplicate. *DEGs* differentially expressed genes

Guided by the inflammasome findings, we profiled upstream pathways using unbiased transcriptomics with pathway enrichment. RNA-seq of nasal mucosa revealed extensive transcriptional changes in AR that were reversed by LFB, with clear clusters of up- and downregulated genes (Fig. 3A, B). Pathway analysis prioritized immune-inflammatory programs, with the PI3K–AKT pathway emerging as a top KEGG term (Fig. 3C), alongside GO categories linked to leukocyte activation, cytokine production and extracellular matrix organization (Fig. 3D). Consistent with these predictions, immunoblotting showed increased phosphorylation of PI3K, AKT and p65 in AR tissue that was markedly reduced after LFB treatment, while total PI3K/AKT levels were largely unchanged (Fig. 3E). These data indicate that LFB attenuates AR-associated signaling by restraining the PI3K–AKT/NF-κB pathway, in line with its suppression of NLRP3-mediated inflammation.

### PI3K–AKT reactivation partially attenuates LFB-mediated suppression of cytokines and NLRP3-inflammasome activation in HNEpCs and mast cells

**Fig. 4:**
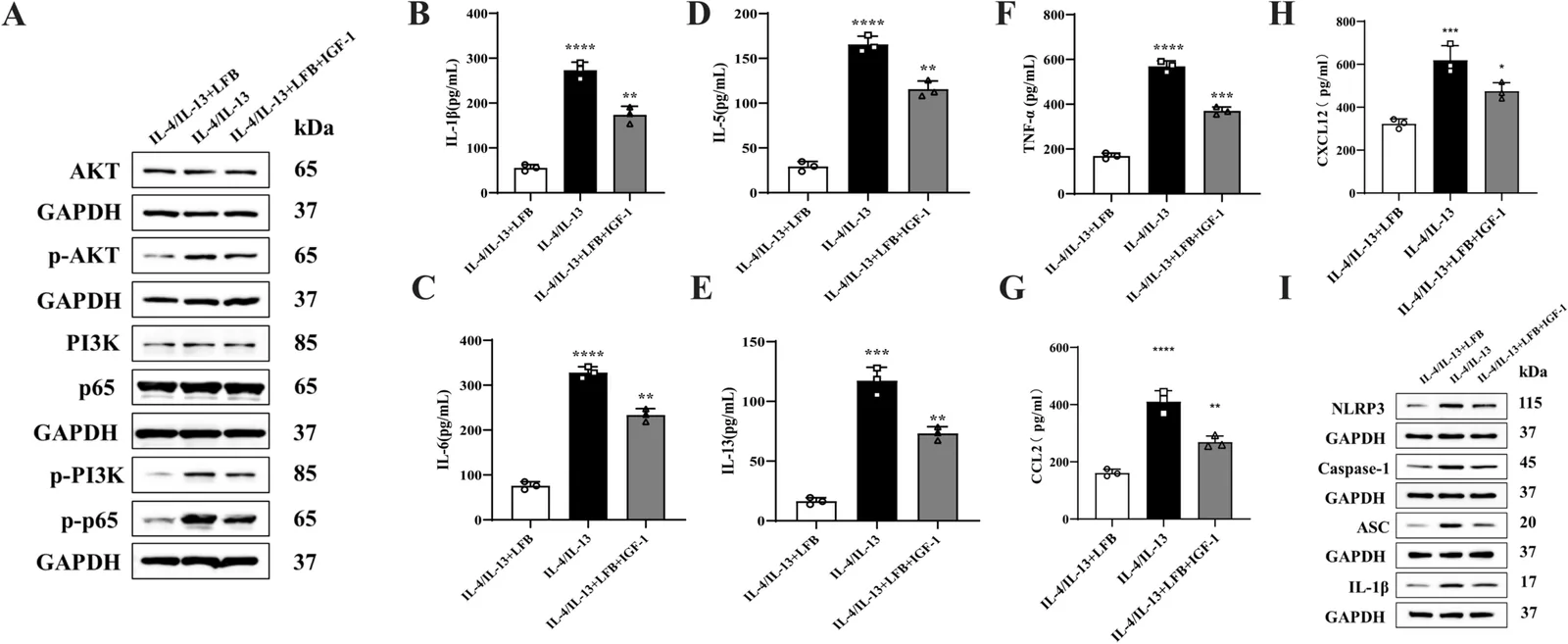
PI3K–AKT activation counteracts the anti-inflammatory effect of LFB. **A** Immunoblots of PI3K–AKT/NF-κB signaling in each group**.** Representative immunoblots were obtained from independent biological experiments performed in triplicate. **B–H** ELISA quantification of IL-1β **(B)**, IL-6 **(C)**, IL-5 **(D)**, IL-13 **(E)**, TNF-α **(F)**, CCL2 **(G)**, and CXCL12 **(H)** in each group**.** Individual data points represent independent biological replicates (n = 3 independent biological samples per group). Data are presented as mean ± SD. **I** Immunoblots of NLRP3-inflammasome components in each group**.** Representative immunoblots were obtained from independent biological experiments performed in triplicate. *P< 0.05, **P< 0.01, ***P< 0.005, ****P< 0.001 versus IL-4/IL-3+LFB

Because PI3K–AKT–NF-κB can license NLRP3 activation, we tested causality by pharmacologically reactivating the pathway in HNEpCs. HNEpCs exhibited typical cobblestone morphology, and exposure to LFB did not reduce cell viability, indicating an acceptable working range for in-vitro assays (Supplementary Fig. S1A,B). Using the LFB group as the reference in stimulated HNEpCs, phosphorylation of PI3K, AKT and p65 was elevated in the AR group and was markedly lowered by LFB; addition of IGF-1 (LFB+IGF-1) re-activated the pathway toward model levels (Fig. 4A). Concordantly, pro-inflammatory cytokines IL-1β, IL-6, IL-5, IL-13 and TNF-α were increased in the model group relative to LFB and were reduced by LFB; IGF-1 partially abrogated these reductions (Fig. 4B–F). Similarly, CCL2 and CXCL12 levels were significantly suppressed by LFB, whereas IGF-1 partially restored their expression (Fig. 4G,H). LFB also diminished NLRP3 inflammasome proteins (NLRP3, ASC, caspase−1 and IL-1β), whereas IGF-1 restored their abundance (Fig. 4I).

To further investigate whether mast cells are directly involved in the anti-inflammatory effects of LFB, activated mast cells were treated with LFB in the presence or absence of IGF-1. ELISA analysis demonstrated that activated mast cells produced significantly higher levels of IL-1β, IL-5, IL-6, IL-13, and TNF-α compared with controls, whereas LFB markedly reduced these cytokines (Supplementary Fig. S2B–F). Importantly, IGF-1 partially reversed the suppressive effects of LFB on inflammatory cytokine production. Consistently, western blot analysis showed that activated mast cells displayed increased expression of NLRP3 inflammasome-related proteins, including NLRP3, ASC, caspase−1, and IL-1β, all of which were suppressed by LFB treatment (Supplementary Fig. S2G). In parallel, phosphorylation of PI3K, AKT, and NF-κB p65 was elevated in activated mast cells and was reduced by LFB, whereas IGF-1 partially restored pathway activation (Supplementary Fig. S2H). These findings suggest that LFB reduces inflammatory activation in stimulated RBL-2H3 mast cells and that PI3K–AKT/NF-κB/NLRP3 signaling may be involved in this effect.

### IGF-1 or poly(I) partially attenuates the symptomatic and histologic improvements induced by LFB

**Fig. 5:**
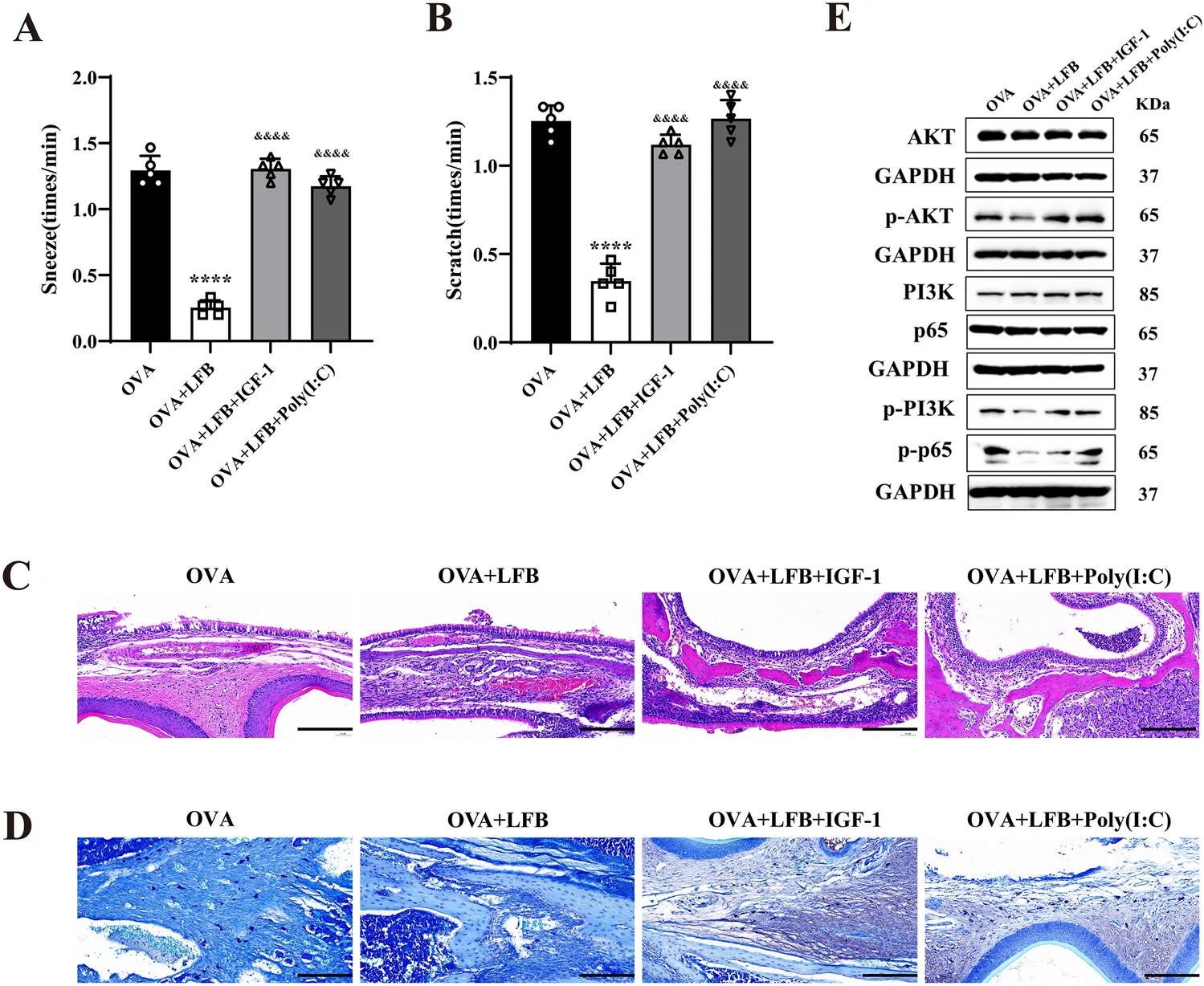
Reactivating PI3K–AKT/NF-κB signaling counteracts LFB’s benefits in allergic rhinitis. **A** Sneezing frequency in each group. **B** Nasal scratching frequency in each group. Individual data points represent independent biological replicates (*n* = 5 mice per group). Data are presented as mean ± SD. **C** Representative H&E staining of nasal mucosa in each group. Scale bar = 50 μm **D** Toluidine blue staining for mast cells in each group. Scale bar = 50 μm. Representative histological images were obtained from independent animals (*n* = 5 mice per group). **E** Immunoblot analysis of PI3K–AKT/NF-κB pathway-related proteins in nasal tissues. Representative immunoblots were obtained from independent biological experiments performed in triplicate. *P< 0.05, **P< 0.01, ***P< 0.005, ****P< 0.001 versus OVA. ^&^*P*< 0.05, ^&&^*P*< 0.01, ^&&&^*P*< 0.005 versus OVA+LFB

To evaluate whether pathway or innate immune reactivation can negate LFB’s efficacy in vivo, we challenged LFB-treated AR mice with IGF-1 or Poly(I:C). Relative to AR, LFB markedly reduced sneezing and nasal scratching (Fig. 5A,B) and improved mucosal architecture on H&E with diminished mast-cell accumulation on toluidine blue (Fig. 5C,D). These benefits were reversed when PI3K–AKT was reactivated by IGF-1 or when inflammation was re-stimulated with Poly(I:C), which increased symptom scores and restored epithelial thickening and mast-cell infiltration toward AR levels (Fig. 5A–D). Consistently, phosphorylated PI3K, AKT and p65 were elevated in AR, suppressed by LFB, and re-elevated by IGF-1 or Poly(I:C), while total PI3K/AKT remained largely unchanged (Fig. 5E). These data suggest that reduced PI3K–AKT/NF-κB activation is associated with the protective effects of LFB in the OVA-induced allergic rhinitis model, and that pathway reactivation by IGF-1 or inflammatory restimulation by poly(I:C) partially attenuates these effects.

### LFB-associated reductions in cytokine output and NLRP3 activation are partially attenuated by IGF-1 or poly(I)

**Fig. 6:**
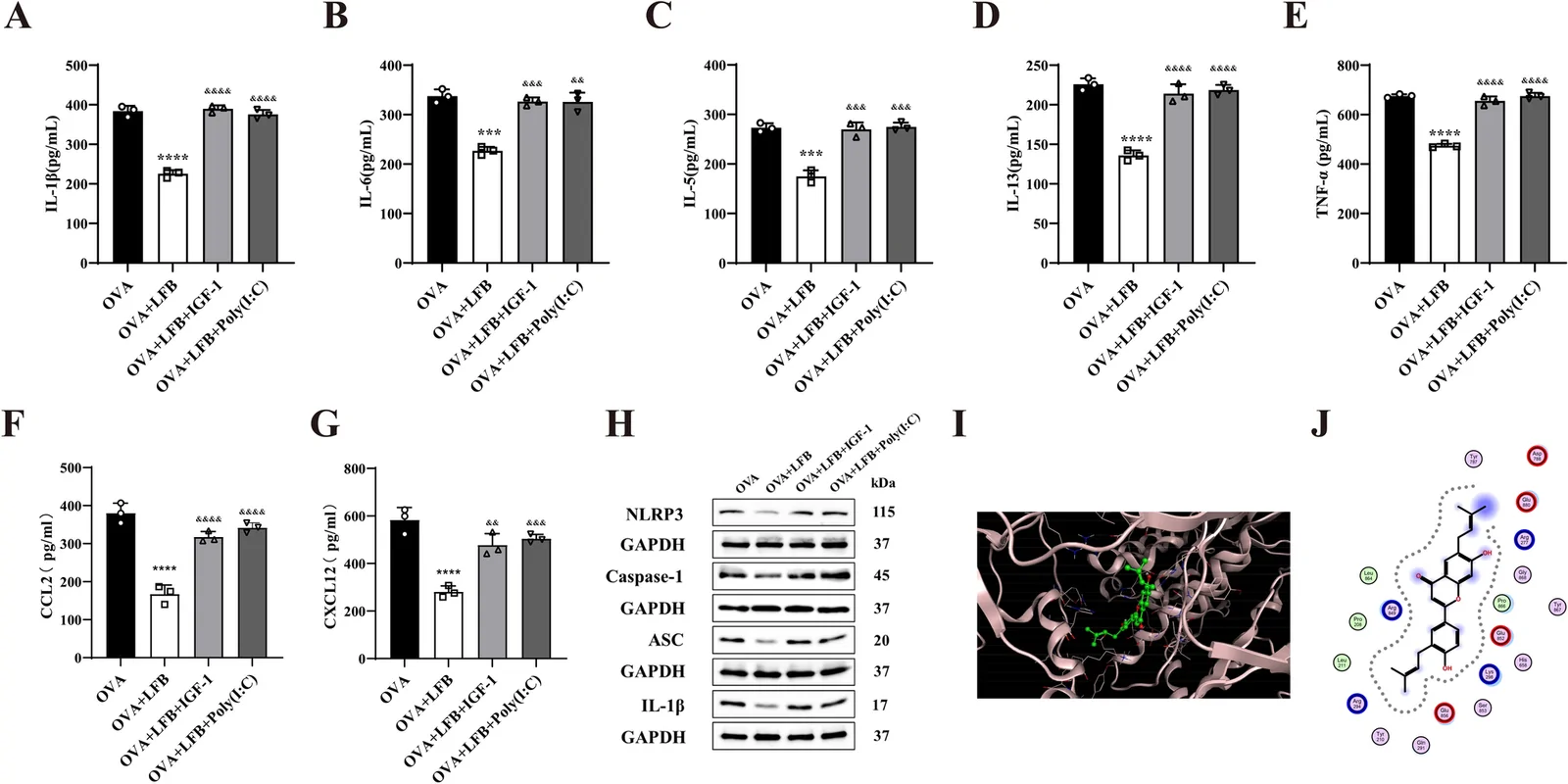
LFB lowers cytokines and NLRP3 through PI3K–AKT/NF-κB. **A–G** ELISA quantification of IL-1β **(A)**, IL-6 **(B)**, IL-5 **(C)**, IL-13 **(D)**, TNF-α **(E)**, CCL2 **(F)**, and CXCL12 **(G)** in OVA, OVA +LFB, OVA+LFB+IGF-1 and OVA +LFB+Poly(I:C) groups. Individual data points represent independent biological replicates (*n* = 3 independent biological samples per group). Data are presented as mean ± SD. **H** Immunoblot analysis of NLRP3-inflammasome components in nasal tissues. Representative immunoblots were obtained from independent biological experiments performed in triplicate. **I, J** In-silico docking models of LFB with PI3K [3D pose (**I)** and 2D interaction **(J)** schematic]. *P< 0.05, **P< 0.01, ***P< 0.005, ****P< 0.001 versus OVA. ^&^*P*< 0.05, ^&&^*P*< 0.01, ^&&&^*P*< 0.005 versus OVA+LFB

Relative to AR, LFB markedly decreased IL-1β, IL-6, IL-5, IL-13 and TNF-α (Fig. 6A–E), as well as CCL2 and CXCL12 levels (Fig. 6F,G), and reduced NLRP3, ASC, caspase−1 and IL-1β abundance (Fig. 6H). Reactivation of signaling with IGF-1 or Poly(I:C) partially restored cytokine production and inflammasome proteins toward AR levels. Consistent with the pathway data, molecular docking positioned LFB within the PI3K catalytic pocket with stable predicted contacts (Fig. 6I, J), providing structural plausibility for a potential interaction between LFB and PI3K. However, this in silico result should be interpreted as hypothesis-generating rather than definitive evidence of direct PI3K inhibition.

## Discussion

The primary finding of the present study is that LFB alleviated OVA-induced allergic rhinitis-like phenotypes and was associated with reduced inflammatory cytokine production, decreased mast-cell staining, and suppression of NLRP3 inflammasome-related proteins in nasal tissues. In vivo, LFB reduced sneezing and nasal scratching, partially improved epithelial thickening and inflammatory infiltration, and decreased toluidine blue-positive mast-cell accumulation. These findings indicate that LFB exerts anti-inflammatory effects at the phenotypic and tissue levels in the OVA-induced allergic rhinitis model. These observations are consistent with the recognized contribution of inflammasome-linked cytokine signaling to airway inflammation and mucosal immune responses [19].

Mechanistically, our experimental evidence mainly supports the involvement of epithelial and mast-cell-associated PI3K–AKT/NF-κB/NLRP3 signaling. In HNEpCs and stimulated RBL-2H3 mast cells, LFB reduced PI3K–AKT/NF-κB activation and inflammatory mediator production, whereas IGF-1 partially reversed these effects. However, macrophage polarization and eosinophil responses were not directly examined in the present study. Therefore, the current data do not allow us to determine whether LFB directly regulates macrophages, eosinophils, or other immune-cell populations in vivo. These issues warrant further investigation in future studies using immune-cell-specific markers and cell-resolved analytical approaches.

At the mediator level, LFB lowered IL-1β, IL-6, IL-5, IL-13, TNF-α, CCL2, and CXCL12 and reduced NLRP3, ASC, caspase−1, and IL-1β abundance in nasal tissues. Transcriptomic analysis prioritized PI3K–AKT among enriched pathways, and immunoblotting showed decreased phosphorylation of PI3K, AKT, and p65 after LFB treatment, while total PI3K and AKT levels remained largely unchanged. In HNEpCs and stimulated RBL-2H3 mast cells, IGF-1 partially restored pathway phosphorylation, inflammatory mediator production, and inflammasome-related protein expression. These findings support the involvement of PI3K–AKT/NF-κB/NLRP3 signaling in the anti-inflammatory effects of LFB, but they do not establish direct PI3K inhibition by LFB.

These observations align with and extend several strands of prior work. First, an accumulating body of evidence links NLRP3 activation to nasal epithelial dysfunction and type−2-skewed inflammation in clinical specimens and preclinical rhinitis models [20, 21]; reduced NLRP3 components in the presence of symptom and histology improvement is directionally consistent with this literature. Second, epithelial PI3K–AKT–NF-κB signaling is a recognized integrator of innate triggers [22]; TLR3 activation with poly(I:C) robustly induces epithelial cytokines relevant to rhinitis and rhinosinusitis, and PI3K inhibition mitigates inflammatory outputs in airway models. In addition to the epithelial PI3K–AKT/NF-κB pathway examined in the present study, lysophospholipid-related signaling and LPA receptor pathways may represent upstream regulatory networks that contribute to inflammatory-cell recruitment and polarization in allergic airway inflammation. However, lysophospholipid species, LPA receptors, macrophage polarization, and eosinophil responses were not directly measured in this study. Therefore, these pathways should be interpreted as plausible future research directions rather than explanatory mechanisms for the present findings. Future studies integrating lipidomic profiling, LPA receptor analysis, macrophage and eosinophil markers, flow cytometry, immunofluorescence, or single-cell sequencing will be required to determine whether these pathways contribute to the broader immunomodulatory effects of LFB in vivo. Consistent with this pathway-based interpretation, PI3K–AKT reactivation by IGF-1 and inflammatory restimulation by poly(I:C) partially attenuated the protective effects of LFB in the present study, supporting the involvement of PI3K–AKT/NF-κB-associated inflammatory signaling without establishing direct PI3K inhibition by LFB.

Third, natural-product flavonoids—including licorice-derived species—have repeatedly been shown to suppress PI3K–AKT [23] and NF-κB signaling [24] across epithelial and myeloid systems, albeit with variable specificity; the present dataset provides pathway-resolved evidence in nasal mucosa and primary HNEpCs, addressing a gap in the rhinitis literature. Of note, LFB has been reported to protect barrier function and dampen inflammation in colitis models [25], offering external plausibility for epithelial-centric effects while not assuming equivalence across tissues.

From a field perspective, the contribution is twofold. Conceptually, the data position a defined licorice flavonoid within a mechanistic cascade—PI3K–AKT/NF-κB licensing upstream of NLRP3—linking epithelial signaling to mucosal inflammation and symptoms. Practically, the reversal of LFB effects by IGF-1 or poly(I:C) provides pathway dependency that is often missing in phytochemical reports, refining the target space for subsequent medicinal chemistry or combination strategies [26].

Molecular docking suggested that LFB may fit within the PI3K catalytic pocket, providing structural plausibility for a potential LFB–PI3K interaction. However, docking is predictive in nature and cannot demonstrate direct target engagement or enzymatic inhibition. Therefore, the docking results should be interpreted as hypothesis-generating and will require validation by biochemical kinase assays, biophysical binding studies, cellular thermal shift assays, or pull-down experiments. Taken together, the present findings support an association between LFB treatment and reduced PI3K–AKT/NF-κB/NLRP3 pathway activation, while the direct molecular target of LFB and the immune-cell-specific mechanisms underlying its in vivo effects remain to be further clarified.

## Supplementary Information

Below is the link to the electronic supplementary material.

Supplementary file1 (DOCX 393 KB)

Supplementary file2 (DOCX 369 KB)

## Data Availability

The datasets and supporting materials generated during and/or analyzed during the current study are available from the corresponding author on reasonable request.
