# Supplementary material for: Licoflavone B mitigates allergic rhinitis by restraining epithelial PI3K–AKT/NF-κB NLRP3 inflammasome signaling
Source: Hum Cell. 2026 Nov 5;39(11):154. doi: 10.1007/s13577-026-01456-3 (PMC13739966; doi:10.1007/s13577-026-01456-3)
Supplement: Supplementary file 1 [file 13577_2026_1456_MOESM1_ESM.docx]

**Supplementary Figure S1. Primary HNEpCs and LFB safety**

(A) Representative phase-contrast image of HNEpCs. Scale bar, 200 μm. (B) Cell-viability assay of HNEpCs exposed to LFB (10–50 μM) shows no measurable cytotoxicity over 48 h. HNEpCs: primary human nasal epithelial cells.

**Supplementary Figure S2. LFB suppresses inflammatory activation, NLRP3 inflammasome signaling, and PI3K–AKT/NF-κB pathway activation in mast cells.**

**(A)** Representative morphology of cultured mast cells. **(B–F)** ELISA analysis of IL-1β, IL-5, IL-6, IL-13, and TNF-α levels in control, activated mast cells, activated mast cells treated with LFB, and activated mast cells treated with LFB+IGF-1. Individual data points represent independent biological replicates (n = 3 independent biological samples per group). Data are presented as mean ± SD. **(G)** Western blot analysis of NLRP3 inflammasome-related proteins (NLRP3, ASC, caspase-1, and IL-1β). **(H)** Western blot analysis of PI3K–AKT/NF-κB signaling pathway proteins, including PI3K, p-PI3K, AKT, p-AKT, p65, and p-p65. Representative immunoblots were obtained from independent biological experiments performed in triplicate. Data are presented as mean ± SD. * indicates comparison vs Control; & indicates comparison vs activated mast cells.
